# 2-[({2-[(2-Hy­droxy-5-meth­oxy­benzyl­idene)amino]­eth­yl}imino)­meth­yl]-4-meth­oxy­phenol

**DOI:** 10.1107/S1600536812018405

**Published:** 2012-05-16

**Authors:** Ali Ourari, Lotfi Baameur, Sofiane Bouacida, Kamel Ouari

**Affiliations:** a Laboratoire d’Electrochimie, d’Ingénierie Moléculaire et de Catalyse Redox (LEIMCR), Faculté des Sciences de l’Ingénieur, Université Farhat Abbas, Sétif 19000, Algeria; b Unité de Recherche de Chimie de l’Environnement et Moléculaire Structurale (CHEMS), Université Mentouri-Constantine, 25000 Algeria

## Abstract

The asymmetric unit of the title compound, C_18_H_20_N_2_O_4_, contains one-half mol­ecule with an inversion center located at the centroid of the mol­ecule. In the crystal, mol­ecules are linked by C—H⋯π inter­actions, forming layers parallel to (101). An intra­molecular O—H⋯N hydrogen bond also occurs.

## Related literature

For the synthesis of similar compounds see: Srinivasan *et al.* (1986 ▶); Moutet & Ourari (1997 ▶); Ourari *et al.* (2008 ▶).
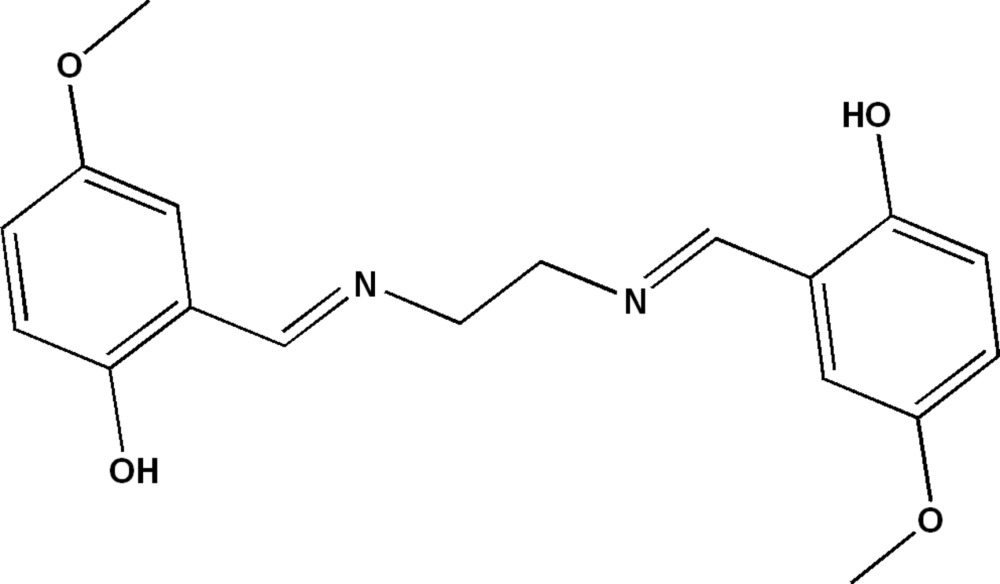


## Experimental

### 

#### Crystal data

C_18_H_20_N_2_O_4_
*M* *_r_* = 328.36
Monoclinic, 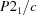

*a* = 15.0040 (12) Å
*b* = 5.9722 (3) Å
*c* = 9.3128 (8) Å
β = 92.001 (3)°
*V* = 833.98 (11) Å^3^
*Z* = 2
Mo *K*α radiation
μ = 0.09 mm^−1^
*T* = 295 K
0.50 × 0.23 × 0.19 mm

#### Data collection

Nonius KappaCCD diffractometer
3001 measured reflections
1664 independent reflections
1097 reflections with *I* > 2σ(*I*)
*R*_int_ = 0.021

#### Refinement

*R*[*F* ^2^ > 2σ(*F* ^2^)] = 0.054
*wR*(*F* ^2^) = 0.167
*S* = 1.05
1664 reflections
111 parameters
H-atom parameters constrained
Δρ_max_ = 0.23 e Å^−3^
Δρ_min_ = −0.16 e Å^−3^

### 

Data collection: *COLLECT* (Nonius, 1998 ▶); cell refinement: *SCALEPACK* (Otwinowski & Minor, 1997 ▶); data reduction: *DENZO* (Otwinowski & Minor 1997 ▶) and *SCALEPACK*; program(s) used to solve structure: *SIR2002* (Burla *et al.*, 2005 ▶); program(s) used to refine structure: *SHELXL97* (Sheldrick, 2008 ▶); molecular graphics: *ORTEP-3 for Windows* (Farrugia, 1997 ▶) and *DIAMOND* (Brandenburg & Berndt, 2001 ▶); software used to prepare material for publication: *WinGX* (Farrugia, 1999 ▶).

## Supplementary Material

Crystal structure: contains datablock(s) global, I. DOI: 10.1107/S1600536812018405/bq2353sup1.cif

Structure factors: contains datablock(s) I. DOI: 10.1107/S1600536812018405/bq2353Isup2.hkl

Supplementary material file. DOI: 10.1107/S1600536812018405/bq2353Isup3.cml

Additional supplementary materials:  crystallographic information; 3D view; checkCIF report

## Figures and Tables

**Table 1:** Hydrogen-bond geometry (Å, °) *Cg* is the centroid of the C4–C9 ring.

*D*—H⋯*A*	*D*—H	H⋯*A*	*D*⋯*A*	*D*—H⋯*A*
O5—H5⋯N2	0.82	1.85	2.5844 (18)	148
C10—H10*C*⋯*Cg*^i^	0.96	2.64	3.521 (2)	152

Symmetry code: (i) 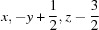.

**Fig. 1.**
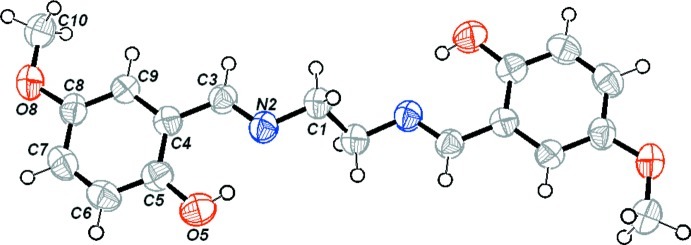
The molecular geometry of (I) with the atom-labeling scheme. Displacement ellipsoids are drawn at the 50% probability level. Only the contents of the asymmetric unit are numbered.

**Fig. 2.**
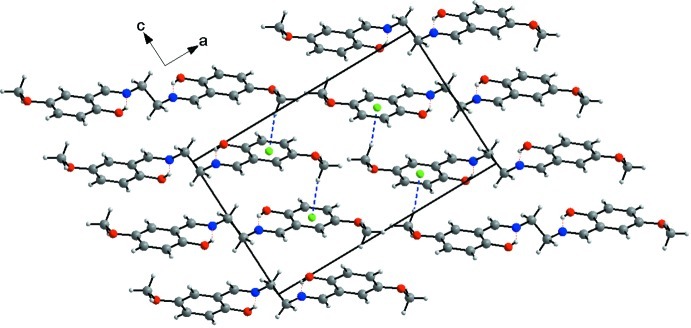
Diagram of layered packing parallel to (101) plane and showing O—H···N and C—H···π interactions.

## supplementary crystallographic information

### Comment

The tetradentate Schiff base ligands derived from salicylaldehyde derivatives and diamino compounds have been found to be excellent chelating agents for most applications in coordination chemistry such as in catalysis (Srinivasan *et al.*, 1986) and electrocatalysis (Moutet & Ourari, 1997; Ourari *et al.*, 2008). Here, we report the synthesis of the title compound and its crystal structure.

The molecular structure of (I), and the atomic numbering used, is illustrated in Fig. 1. The asymmetric unit of the title compound, consists of one-half of the molecule, with the other half generated by a crystallographic inversion center. The crystal packing in the title structure can be described by a zigzag layers parallel to (101) plane (Fig. 2). There is one intramolecular O—H···N hydrogen bonding in this packing (Table 1, Fig. 2), which it is stabilized C—H···π and Van der Walls interactions (table 1) All these interactions link the molecules within the layers and also link the layers together and reinforcing the cohesion of the structure.

### Experimental

60 mg of 1,2-diaminoethane (1 mmol) were dissolved in 10 ml of absolute ethanol. This solution was drop wise added, under stirring, to an ethanolic solution (10 ml) containing 304 mg of 5-methoxysalicylaldehyde (2 mmol). This mixture was refluxed for 1 h after which a yellow precipitate is formed, recovered by filtration, washed several times with diethyl oxide and dried to yield 282 mg (86%) of the title compound. The suitable crystals for X-ray analysis were obtained by slow evaporation from a mixture of solvents ethanol/dichloromethane (8/2, *v*/*v*).

### Refinement

The H atoms were localized on Fourier maps but introduced in calculated positions and treated as riding on their parent atoms (C and O) with C—H = 0.93 Å (methine, aromatic), 0.96 Å (methyl), 0.97 Å (methylene) and O—H = 0.82 Å (hydroxyl) with *U*_iso_(H) = 1.2*U*_eq_(C) or *U*_iso_(H) = 1.5*U*_eq_(O).

### Crystal data

**Table:**

C_18_H_20_N_2_O_4_	*F*(000) = 348
*M**_r_* = 328.36	*D*_x_ = 1.308 Mg m^−^^3^
Monoclinic, *P*2_1_/*c*	Mo *K*α radiation, λ = 0.71073 Å
Hall symbol: -P 2ybc	Cell parameters from 1761 reflections
*a* = 15.0040 (12) Å	θ = 1.0–26.4°
*b* = 5.9722 (3) Å	µ = 0.09 mm^−^^1^
*c* = 9.3128 (8) Å	*T* = 295 K
β = 92.001 (3)°	Prism, colorless
*V* = 833.98 (11) Å^3^	0.50 × 0.23 × 0.19 mm
*Z* = 2	

### Data collection

**Table:**

Nonius KappaCCD diffractometer	1097 reflections with *I* > 2σ(*I*)
Radiation source: Enraf–Nonius FR590	*R*_int_ = 0.021
Graphite monochromator	θ_max_ = 26.4°, θ_min_ = 2.7°
Detector resolution: 9 pixels mm^-1^	*h* = −18→18
CCD rotation images, thick slices scans	*k* = −6→7
3001 measured reflections	*l* = −11→11
1664 independent reflections	

### Refinement

**Table:**

Refinement on *F*^2^	Secondary atom site location: difference Fourier map
Least-squares matrix: full	Hydrogen site location: inferred from neighbouring sites
*R*[*F*^2^ > 2σ(*F*^2^)] = 0.054	H-atom parameters constrained
*wR*(*F*^2^) = 0.167	*w* = 1/[σ^2^(*F*_o_^2^) + (0.101*P*)^2^ + 0.0006*P*] where *P* = (*F*_o_^2^ + 2*F*_c_^2^)/3
*S* = 1.05	(Δ/σ)_max_ < 0.001
1664 reflections	Δρ_max_ = 0.23 e Å^−^^3^
111 parameters	Δρ_min_ = −0.16 e Å^−^^3^
0 restraints	Extinction correction: *SHELXL97* (Sheldrick, 2008), Fc^*^=kFc[1+0.001xFc^2^λ^3^/sin(2θ)]^-1/4^
Primary atom site location: structure-invariant direct methods	Extinction coefficient: 0.08 (2)

### Special details

**Table:**

Geometry. All e.s.d.'s (except the e.s.d. in the dihedral angle between two l.s. planes) are estimated using the full covariance matrix. The cell e.s.d.'s are taken into account individually in the estimation of e.s.d.'s in distances, angles and torsion angles; correlations between e.s.d.'s in cell parameters are only used when they are defined by crystal symmetry. An approximate (isotropic) treatment of cell e.s.d.'s is used for estimating e.s.d.'s involving l.s. planes.
Refinement. Refinement of *F*^2^ against ALL reflections. The weighted *R*-factor *wR* and goodness of fit *S* are based on *F*^2^, conventional *R*-factors *R* are based on *F*, with *F* set to zero for negative *F*^2^. The threshold expression of *F*^2^ > σ(*F*^2^) is used only for calculating *R*-factors(gt) *etc*. and is not relevant to the choice of reflections for refinement. *R*-factors based on *F*^2^ are statistically about twice as large as those based on *F*, and *R*- factors based on ALL data will be even larger.

### Fractional atomic coordinates and isotropic or equivalent isotropic displacement parameters (Å^2^)

**Table:**

	*x*	*y*	*z*	*U*_iso_*/*U*_eq_	
C1	0.00544 (12)	0.5332 (3)	0.42227 (18)	0.0554 (5)	
H1A	0.0237	0.404	0.3674	0.066*	
H1B	−0.0511	0.586	0.3816	0.066*	
C3	0.13689 (11)	0.6835 (3)	0.32973 (18)	0.0500 (5)	
H3	0.1399	0.5537	0.2749	0.06*	
C4	0.20642 (11)	0.8520 (3)	0.31789 (17)	0.0488 (5)	
C5	0.20521 (13)	1.0483 (3)	0.40154 (19)	0.0545 (5)	
C6	0.27360 (14)	1.2037 (3)	0.3894 (2)	0.0620 (5)	
H6	0.2731	1.3341	0.4439	0.074*	
C7	0.34154 (14)	1.1672 (3)	0.2983 (2)	0.0623 (5)	
H7	0.3863	1.2739	0.2909	0.075*	
C8	0.34469 (12)	0.9719 (3)	0.21605 (18)	0.0542 (5)	
C9	0.27715 (11)	0.8166 (3)	0.22553 (18)	0.0515 (5)	
H9	0.2784	0.687	0.1702	0.062*	
C10	0.42592 (14)	0.7497 (3)	0.0532 (3)	0.0764 (7)	
H10A	0.4313	0.6263	0.119	0.115*	
H10B	0.4781	0.7572	−0.0033	0.115*	
H10C	0.3742	0.7287	−0.0089	0.115*	
N2	0.07208 (9)	0.7091 (2)	0.41315 (16)	0.0544 (5)	
O5	0.13958 (10)	1.0889 (2)	0.49361 (15)	0.0712 (5)	
H5	0.1037	0.9853	0.4903	0.107*	
O8	0.41724 (10)	0.9514 (2)	0.13122 (15)	0.0727 (5)	

### Atomic displacement parameters (Å^2^)

**Table:**

	*U*^11^	*U*^22^	*U*^33^	*U*^12^	*U*^13^	*U*^23^
C1	0.0480 (10)	0.0591 (10)	0.0594 (11)	−0.0045 (7)	0.0065 (8)	−0.0027 (8)
C3	0.0467 (10)	0.0539 (9)	0.0494 (10)	0.0010 (7)	0.0029 (8)	−0.0004 (7)
C4	0.0494 (10)	0.0483 (9)	0.0486 (10)	0.0007 (7)	0.0001 (8)	0.0034 (6)
C5	0.0598 (12)	0.0495 (10)	0.0541 (10)	0.0057 (8)	0.0025 (9)	0.0020 (7)
C6	0.0718 (13)	0.0466 (9)	0.0672 (12)	−0.0003 (8)	−0.0038 (10)	−0.0033 (8)
C7	0.0652 (12)	0.0518 (10)	0.0696 (12)	−0.0104 (8)	−0.0025 (10)	0.0063 (9)
C8	0.0517 (11)	0.0589 (10)	0.0521 (10)	−0.0062 (7)	0.0040 (8)	0.0086 (7)
C9	0.0546 (11)	0.0514 (9)	0.0488 (10)	−0.0030 (7)	0.0044 (9)	−0.0013 (7)
C10	0.0628 (13)	0.0883 (14)	0.0793 (15)	−0.0105 (10)	0.0209 (11)	−0.0118 (11)
N2	0.0486 (9)	0.0571 (9)	0.0578 (9)	−0.0012 (6)	0.0068 (7)	−0.0004 (6)
O5	0.0726 (10)	0.0631 (8)	0.0789 (10)	0.0062 (6)	0.0182 (8)	−0.0140 (6)
O8	0.0643 (9)	0.0764 (9)	0.0786 (10)	−0.0194 (7)	0.0215 (7)	−0.0033 (7)

### Geometric parameters (Å, º)

**Table:**

C1—N2	1.455 (2)	C6—H6	0.93
C1—C1^i^	1.515 (3)	C7—C8	1.397 (2)
C1—H1A	0.97	C7—H7	0.93
C1—H1B	0.97	C8—O8	1.373 (2)
C3—N2	1.275 (2)	C8—C9	1.379 (2)
C3—C4	1.456 (2)	C9—H9	0.93
C3—H3	0.93	C10—O8	1.415 (2)
C4—C9	1.405 (2)	C10—H10A	0.96
C4—C5	1.408 (2)	C10—H10B	0.96
C5—O5	1.350 (2)	C10—H10C	0.96
C5—C6	1.391 (3)	O5—H5	0.82
C6—C7	1.366 (3)		
			
N2—C1—C1^i^	109.99 (18)	C6—C7—C8	120.91 (16)
N2—C1—H1A	109.7	C6—C7—H7	119.5
C1^i^—C1—H1A	109.7	C8—C7—H7	119.5
N2—C1—H1B	109.7	O8—C8—C9	125.19 (16)
C1^i^—C1—H1B	109.7	O8—C8—C7	115.64 (15)
H1A—C1—H1B	108.2	C9—C8—C7	119.18 (17)
N2—C3—C4	121.80 (15)	C8—C9—C4	120.69 (16)
N2—C3—H3	119.1	C8—C9—H9	119.7
C4—C3—H3	119.1	C4—C9—H9	119.7
C9—C4—C5	119.26 (16)	O8—C10—H10A	109.5
C9—C4—C3	120.03 (15)	O8—C10—H10B	109.5
C5—C4—C3	120.68 (16)	H10A—C10—H10B	109.5
O5—C5—C6	119.26 (16)	O8—C10—H10C	109.5
O5—C5—C4	121.64 (16)	H10A—C10—H10C	109.5
C6—C5—C4	119.10 (17)	H10B—C10—H10C	109.5
C7—C6—C5	120.86 (16)	C3—N2—C1	119.31 (15)
C7—C6—H6	119.6	C5—O5—H5	109.5
C5—C6—H6	119.6	C8—O8—C10	117.41 (14)
			
N2—C3—C4—C9	179.47 (16)	C6—C7—C8—C9	1.2 (3)
N2—C3—C4—C5	1.4 (3)	O8—C8—C9—C4	178.87 (17)
C9—C4—C5—O5	−178.94 (15)	C7—C8—C9—C4	−0.8 (3)
C3—C4—C5—O5	−0.9 (3)	C5—C4—C9—C8	−0.2 (2)
C9—C4—C5—C6	0.8 (2)	C3—C4—C9—C8	−178.27 (15)
C3—C4—C5—C6	178.82 (15)	C4—C3—N2—C1	−178.95 (15)
O5—C5—C6—C7	179.38 (17)	C1^i^—C1—N2—C3	127.0 (2)
C4—C5—C6—C7	−0.3 (3)	C9—C8—O8—C10	−3.6 (3)
C5—C6—C7—C8	−0.7 (3)	C7—C8—O8—C10	176.06 (17)
C6—C7—C8—O8	−178.45 (17)		

Symmetry code: (i) −*x*, −*y*+1, −*z*+1.

### Hydrogen-bond geometry (Å, º)

Cg is the centroid of the C4–C9 ring.

**Table:**

*D*—H···*A*	*D*—H	H···*A*	*D*···*A*	*D*—H···*A*
O5—H5···N2	0.82	1.85	2.5844 (18)	148
C10—H10*C*···*Cg*^ii^	0.96	2.64	3.521 (2)	152

Symmetry code: (ii) *x*, −*y*+1/2, *z*−3/2.

## References

[bb1] Brandenburg, K. & Berndt, M. (2001). *DIAMOND* Crystal Impact GbR, Bonn, Germany.

[bb2] Burla, M. C., Caliandro, R., Camalli, M., Carrozzini, B., Cascarano, G. L., De Caro, L., Giacovazzo, C., Polidori, G. & Spagna, R. (2005). *J. Appl. Cryst.* **38**, 381–388.

[bb3] Farrugia, L. J. (1997). *J. Appl. Cryst.* **30**, 565.

[bb4] Farrugia, L. J. (1999). *J. Appl. Cryst.* **32**, 837–838.

[bb5] Moutet, J. C. & Ourari, A. (1997). *Electrochim. Acta*, **42**, 2525–2531.

[bb6] Nonius (1998). *COLLECT* Nonius BV, Delft, The Netherlands.

[bb7] Otwinowski, Z. & Minor, W. (1997). *Methods in Enzymology*, Vol. 276, *Macromolecular Crystallography*, Part A, edited by C. W. Carter Jr & R. M. Sweet, pp. 307–326. New York: Academic Press.

[bb8] Ourari, A., Baameur, L., Bouet, G. & Khan, A. M. (2008). *J. Electrochem. Commun.* **10**, 1736–1739.

[bb9] Sheldrick, G. M. (2008). *Acta Cryst.* A**64**, 112–122. 10.1107/S0108767307043930 18156677

[bb10] Srinivasan, K., Michaud, P. & Kochi, J. K. (1986). *J. Am. Chem. Soc.* **108**, 2309–2320. 10.1021/ja00269a029 22175576

